# Editorial: Advances in Management and Treatment of High Myopia and Its Complications

**DOI:** 10.3389/fmed.2022.846540

**Published:** 2022-03-10

**Authors:** Quan V. Hoang, Xavier Chan, Xiangjia Zhu, Xiangtian Zhou, Xiangchao Shentu, Yi Lu

**Affiliations:** ^1^Singapore Eye Research Institute, Singapore National Eye Centre, Duke-NUS, Singapore, Singapore; ^2^Department of Ophthalmology, Yong Loo Lin School of Medicine, National University of Singapore, Singapore, Singapore; ^3^Department of Ophthalmology, Edward S. Harkness Eye Institute, Columbia University College of Physicians and Surgeons, New York, NY, United States; ^4^Eye Institute, Eye & ENT Hospital, Fudan University, Shanghai, China; ^5^School of Optometry and Ophthalmology and Eye Hospital, Wenzhou Medical University, Wenzhou, China; ^6^Department of Ophthalmology, The Second Affiliated Hospital of Zhejiang University, College of Medicine, Hangzhou, China

**Keywords:** high myopia (HM), pathologic myopia, refractive surgery, cataract surgery, highly myopic cataract (HMC), myopic traction maculopathy (MTM), myopic macular degeneration, myopic choroidal neovascularization (mCNV)

High myopia (HM), or extreme near-sightedness, is a leading cause of blindness worldwide. By 2050, it is estimated that 4.8 billion people will have myopia worldwide, of which almost a billion will have HM (1). HM is due to progressive, lifelong and extreme eye elongation with subsequent eye wall (sclera) thinning; which may lead to localized, posterior eye shape changes (focal, ectatic outpouchings called *staphyloma*), which often precede or are concurrent with the onset of degenerative myopic macular degeneration (MMD) (2, 3). The main threats to vision in a HM patient are the development of MMD, staphyloma and macular traction maculopathy (MTM), and myopic choroidal neovascularization (mCNV) (4–8). The treatment options for the non-neovascular posterior segment manifestations of PM are scarce, but include novel approaches to achieve posterior scleral reinforcement such as with macular buckles, scleral allografts and various scleral collagen crosslinking agents (2, 8). In terms of anterior segment changes, cataract surgery and refractive surgeries in HM eyes has less predictable outcomes vs. emmetropic eyes, and require more careful pre-operative assessment and planning. Over the past decades, several groups of clinicians and scientists have investigated the pathologies, governing mechanisms, diagnostic and therapeutic options for high myopia and documented complications in tandem. In this issue of Frontiers in Medicine, advancements in the management and treatment of both the posterior and anterior segment manifestations of high myopia (HM) were investigated.

In terms of posterior segment manifestations, pathologic myopia (PM) tends to come in three main forms, the degenerative myopic atrophy maculopathy (MAM, including MMD), the tractional MTM, and the neovascular myopic neovascular maculopathy (or mCNV). Tey et al. demonstrated in the Myopic and Pathologic Eyes in Singapore (MyoPES) cohort that greater prevalences of degenerative forms of pathologic myopia (PM) occurred in eyes longer than 27.5 mm and prevalences of tractional forms of PM were greater in eyes longer than 29.0 mm (Ky et al.). Tian et al. investigated the association between the tractional and degenerative forms of PM in eyes and found that over 72% of their cohort with definite myopic retinoschisis also displayed diffuse chorioretinal atrophy.

In terms of degenerative forms of PM, Shi et al. employed non-invasive quantitative and qualitative measurements of retinal and choroidal microvasculature using optical coherence tomographic angiography (OCTA) and found that a longer baseline AL was associated with larger changes of macular vessel density in the inner-inferior, inner-temporal and outer-temporal sectors (Shi et al.). Specifically, on swept source OCTA, in a Singaporean case-control study, Wong et al. report lower macular vessel density (VD) and smaller superficial FAZ area were found in adolescent and young adults with HM compared to those without. Moreover, in a cross-sectional, population-based study of young adults focused on fundus tessellation, Lyu et al. report that tessellation was found to be significantly correlated with AL, scleral thinning and choroidal thinning (particularly in the macula-papillary region). Early indicators for choroidal thickness in young myopic patients were also investigated by Sun et al. who used a machine learning approach with quantifiable models of imaging features and early changes of optic disc and peripapillary region were found to be significantly correlated with choroidal thickness. Meng et al. observed in a retrospective study of 1,692 patients that cilioretinal arteries were found to be associated with MMD, and proposed a photographic classification system and suggested cilioretinal arteries may afford a protective effect (e.g., better visual acuity) when present in HM eyes in a retrospective study.

In patients who develop mCNV, Xie et al. performed a study to assess the relationship between mCNV presence with choroidal thickness and scleral thickness. Intriguingly, over 78% of their cohort of 88 mCNV eyes had nearby scleral perforated vessels detected. For mCNV eyes undergoing treatment with anti-vascular endothelial growth factor (VEGF), Wang, Hu, et al. used an OCTA-based analysis and highlighted vessel junctions as a potential predictive biomarker for early therapeutic response to anti-VEGF therapy.

The treatment options for the non-neovascular posterior segment manifestations of PM are scarce. In terms of posterior scleral reinforcement (PSR) of the posterior pole, Zhang et al. reported short-term improvements in choroidal thickness and choroidal blood flow 1 month post-PSR. Recently, a novel approach to target the sclera by weakening, thinning, and expanding the sclera *via* scleral collagen cross-linking (SXL) have been proposed to halt the progression of myopia, thereby preventing aberrant scleral remodeling (Wang, Corpuz, et al.). For patients who develop myopic foveoschisis (MF) with foveal detachment, Yao et al. employed pars plana vitrectomy with silicone oil (SO) tamponade but without internal limiting membrane peeling, which was found to result in complete MF resolution and foveal re-attachment with no macular hole formation in a case series of 3 patients.

In terms of advancements in the management of anterior segment manifestations of HM, Zhao et al. reported greater variation in peripheral ACD and anterior chamber angle (ACA) after toric vs. non-toric implantable collamer lenses (ICL) and suggest that pre-operative anterior chamber structure and value affect postoperative peripheral ACD and ACA. Moreover, He et al. reported that risk of low vision post-cataract surgery, when treatment was performed by a junior surgeon, is greater in eyes with known macular complications, higher corneal astigmatism, longer axial length and thinner subfoveal choroidal thickness. In an attempt to improve the accuracy of intraocular lens (IOL) power prediction for cataract surgery with IOL implantation in HM eyes, Wei et al. developed a machine learning-based XGBoost calculator in order to improve the accuracy of IOL power predictions in highly myopic cataract (HMC) patients. Zhu et al. demonstrated that among their cohort of 142 HM eyes with cataracts, those with anterior chamber depth (ACD) > 3 mm benefited from femtosecond laser capsultomy in terms of superior capsulorrhexis sizing and long-term IOL centration.

In summary, this issue on Advances in Management and Treatment of High Myopia and Its Complications highlights advancements in the management and treatment of both the posterior and anterior segment manifestations of high myopia. While several novel approaches have been proposed, findings will undoubtedly need to be further validated before widespread adoption.

## Author Contributions

QH and XC drafted the manuscript. QH, XC, XZhu, XZhou, XS, and YL critically proofread and edited the manuscript. All authors contributed to the article and approved the submitted version.

## Conflict of Interest

The authors declare that the research was conducted in the absence of any commercial or financial relationships that could be construed as a potential conflict of interest.

## Publisher's Note

All claims expressed in this article are solely those of the authors and do not necessarily represent those of their affiliated organizations, or those of the publisher, the editors and the reviewers. Any product that may be evaluated in this article, or claim that may be made by its manufacturer, is not guaranteed or endorsed by the publisher.
